# Supplementary material for: Food and Nutrient Intake during Pregnancy in Relation to Maternal Characteristics: Results from the NICE Birth Cohort in Northern Sweden
Source: Nutrients. 2019 Jul 22;11(7):1680. doi: 10.3390/nu11071680 (PMC6682885; doi:10.3390/nu11071680)
Supplement: Supplementary file 1 [file nutrients-11-01680-s001.pdf]

nutrients

MDPI

Supplementary file S1: Modifications made to the dietary questionnaire Meal-Q

1. Modification of the nutrition calculation program to distinguish between the different fat contents of "Milk, sour milk or yogurt" and "Other milk products, e.g., cream or crème fraiche", when the consumption patterns of these food items were requested, as well as when there were follow-up questions as to whether they usually chose low calorie products.

2. Addition to the adult questionnaire of questions regarding game meat and lamb, fish species before and during pregnancy, organic food purchases, and consumption of gluten and lactose. These questions were not included in the nutrition calculation.

3. Addition of a follow-up question to those respondents who reported in the adult questionnaire that they eat flaxseeds, with corresponding modification of the nutrition calculation program (i.e., different nutritional contents of crushed and whole flaxseeds, respectively).

4. Addition to the adult survey of questions regarding the participants' consumption patterns of products with probiotics. This does not affect the nutrition calculation program.

5. Modification of the nutrition calculation program to distinguish between sweet and "low-calorie" drinks.

Supplementary file S2: Estimated sizes of normal portions

Estimated normal portions

If there was a substantial difference in the definition of a normal portion across the food items included in the same variable, a mean value for the normal portions was taken.

Search terms in the national food database (The Swedish National Food Agency)

Food item in questionnaire	Normal portion (in grams)	Search term in the SNFA database
Bread (white)	30	Bröd vitt fibrer ca 5% typ Jättefranska
Bread (whole-grain)	40	Rågbröd fullkorn fibrer ca 6% typ Lingongrova Skördeglädje Gott gräddat
Rice	180	Ris långkornigt kokt m salt
Pasta	170	Pasta berikad kokt m salt
Bulgur	140	Bulgur kokt
Hard rye bread	10	Hårt bröd fullkorn typ vanligt knäckebröd
Porridge	275	Gröt fullkorn ospec.
Egg	50	Ägg konventionellt kokt
Fat on sandwich	5	Matfettsblandning fett 75% berikad typ Bregott
Breakfast cereals	40	Frukostflingor flingblandning fullkorn Basmüsli
Jam	20	Jordgubbssylt
Cheese	15	Ost hårdost fett 31%
Ham (on sandwich)	15	Gris skinka fett 1-3% skivad rökt
Ground meat dishes	100<br>150<br>350<br>200	Köttbullar fett ca 15% stekta frysta<br>Köttfärssås blandfärs<br>Lasagne
Dressing with mayonnaise	25	Dressing majonäs fett ca 40%
Ketchup	12	Ketchup
Salmon	130	Lax stekt
Cod	125	Torsk filé kokt
Fish balls	175	Fiskbullar m hummersås konserv tillagad
Shrimps	100	Räkor kokta
Soup	250	Fisksoppa
Pizza	350	Pizza Capricciosa rökt skinka restaurang
Pancakes	225	Pannkaka tunn hemlagad
Pommes frites	150	Pommes frites friterad potatis fett ca 11% fryst
Potatoes	175	Potatis kokt m salt
Milk in coffee	10	Kaffegrädde fett 12%
Coffee	150	Kaffe bryggt
Lemonade	330	Läsk m kolsyra
Mayonnaise	40	Majonnäs fett 80%
Cream sauce	40	Gräddfils- o crème fraichesås
Vinaigrette	15	Vinägrett dressing fett 45%
Beans	190	Vita bönor torkade kokta m salt
Olives	5	Oliver gröna m paprikafyllning avrunna
Roots	83	Palsternacka kokt
Green peas	100	Gröna ärter kokta
Broccoli	100	Broccoli kokt
Avocado	55	Avokado

Corn	100	Majskorn konserv u lag
Carrot	70	Morot
Onion	20	Lök gul
Spinach	30	Spenat färsk
Lettuce/cucumber/squash	50	Gurka
Tomato	50	Tomat
Vegetable mix	100	Wokgrönsaker Classic wokade i rapsolja
Brown rice	120	Råris fullkorn långkornigt kokt m salt
Whole-grain pasta	155	Pasta >50% fullkorn kokt m salt
Baguette/wrap	475<br>250<br>362.5	Baguette grov m ost skinka sallad lättmajonnäs<br>Wrap vetetortilla m fetaost oliver sallad<br>vitlöksdressing
Salad (total)	100	Grönsallat gurka tomat paprika u dressing
Vegetarian protein source	17<br>100<br>100<br>72.3	Kikärtsbiffar falafel friterade<br>Sojakorv stekt<br>Quorn svampprotein färs bitar filé
Vegetarian carbohydrate source	350<br>150<br>250	Lasagne vegetarisk spenat chèvre<br>Gratäng grönsaker rotfrukter
Herring/salmon/trout from the Baltic Sea	35<br>130<br>125<br>96.7	Strömming filé stekt<br>Lax stekt<br>Öring odlad rå
Perch/pike/pike-perch/turbot (lake fish)	125<br>125	Gädda panerad stekt<br>Abborre filé panerad stekt
Rainbow trout (lake fish)	125	Regnbågslax panerad stekt
Tuna	94	Tonfisk stekt
Hare	100	Hare stek stekt
Wild boar	125	Vildsvin kött rå
Deer	100	Rådjur kött ugnsstekt
Elk	125	Älgfärsbiff stekt
Reindeer	100	Ren kött kokt
Lamb	100	Lamm kotlett stekt
Liver	70<br>125<br>115<br>103.3	Gris lever tillagad<br>Kyckling lever tillagad<br>Nöt lever stekt
Blood pudding/sausage	150	Blodpudding blodkorv fett 13% stekt
Pork	84	Gris fläsk kotlett stekt
Bacon	50	Bacon gris rökt stekt
Beef	85	Nöt biff ryggbiff stekt
Sausage	50<br>100<br>100<br>83.3	Varmkorv stekt<br>Wienerkorv kokt<br>Falukorv stekt
Chicken	125	Kyckling bröst m skinn stekt
Hamburger/kebab/tacos	90<br>425<br>250<br>255	Hamburgare blandfärs stekt<br>Taco m köttfärsfyllning m.m.<br>Kebab m bröd sallad sås

Tea	250	Te bryggt
Water	250	Kranvatten
Chocolate milk	200	Mjölkdryck jordgubb fett 1.5% berik
Milk (glass or plate)	200	Mellanmjölk fett 1.5% berik m D-vitamin
Juice	200	Juice ospec.
Fruit soup/cream/smoothie	200<br>250<br>125<br>191.7	Fruktdryck nypon<br>Fruktsoppa ätf blandad torkad frukt pastöriserad berik<br>Kräm bär frukt ospec.
Oatmeal drink	200	Havredryck berikad
Nuts	40	Chilinötter
Ice cream	75	Gräddglass fett 12%
Pastries/cake	48<br>110<br>150<br>102.7	Punschrulle Dammsugare<br>Bärtårta grädde vaniljkräm sylt gele<br>Äppelpaj
Bun	25	Fyllt vetebröd bullar längd ospec.
Crackers/biscuits	10	Veteskorpor
Yoghurt	200	Yoghurt naturell fett 3%
Orange	125	Apelsin
Apple	125	Äpple m skal
Liver pâté	14	Leverpastej bredbar skivbar fett ca 24%
Soy drink	200	Sojadryck berikad
Dried fruit	45	Aprikoser torkade
Berries	125	Blåbär
Other fruit (other than banana, apple, orange, citrus or grape)	85<br>150<br>117.5	Kiwi grön<br>Honungsmelon
Banana	105	Banan
Cottage cheese/brie	15<br>100<br>50<br>55	Färskost fett 27% typ Philadelphia<br>Cottage cheese färskost naturell fett 4% typ Keso (R)<br>Brieost vitmögelost fett 30%
Probiotics (yoghurt/fil)	200	A-fil fett 3%
Flaxseed	8	Based on the National recommendation
Popcorn	30	Based on the declaration of ingredients in one of the most common brands in Sweden (OLW)
Chips	30	Based on the declaration of ingredients in one of the most common brands in Sweden (OLW)

Converting range into means for variables with information about amount

Amount answered in the questionnaire	Estimated normal portions (mean values in grams)
Candy (g)	
<50	24.5
50–99	74.5
100–199	149.5

200–299	249.5
≥300	300
Don't know	249.51[1]
Chocolate (g)	
<25	12
25–49	37
50–99	74.5
100–199	149.5
≥200	200
Don't know	82.92[2]

[1]Calculated as: 0+49+50+99+100+199+200+299+300/9.

[2]Calculated as: 0+24+25+49+50+99+100+199+200/9.

Supplementary table S1: Reported dietary intake of fatty acids in gram per day (n=567).

	**Total reported daily intake**		
**Fatty acid (g/day)**	**Mean (SD)**	**Median (IQR)**	**Min-max**
4:0-10:0	2.6 (1.3)	2.4 (1.7-3.3)	0.4-12.2
12:0	1.1 (0.5)	1.1 (0.8-1.4)	0.2-4.2
14:0	3.4 (1.5)	3.2 (2.3-4.3)	0.7-13.7
16:0	15.4 (5.9)	14.9 (11.1-18.6)	3.9-43.4
18:0	7.2 (3.9)	6.4 (4.6-8.6)	1.6-28.5
20:0	0.2 (0.1)	0.2 (0.2-0.3)	0.1-0.9
Total saturated fat	30.9 (12.6)	29.6 (21.7-37.2)	7.4-92.6
16:1 n-7	1.2 (0.4)	1.2 (0.9-1.5)	0.3-3.3
18:1 n-9	22.7 (8.5)	21.5 (16.3-27.3)	5.7-53.0
Total monounsaturated fat	24.9 (9.1)	23.5 (18.1-29.8)	6.3-57.7
18:2 n-6	6.9 (3.4)	6.2 (4.5-8.6)	1.6-25.1
20:4 n-6	0.09 (0.04)	0.09 (0.06-0.11)	0.02-0.27
18:3 n-3	1.4 (0.8)	1.2 (0.9-1.6)	0.3-8.0
20:5 n-3	0.10 (0.08)	0.08 (0.05-0.15)	0.00-0.50
22:5 n-3	0.05 (0.03)	0.05 (0.03-0.08)	0.00-0.24
22:6 n-3	0.21 (0.14)	0.17 (0.11-0.30)	0.01-1.00
Total polyunsaturated fat	9.0 (4.1)	8.1 (6.0-11.3)	2.1-29.3

Supplementary table S2: Reported dietary intake of micronutrients per day (n=567).

	Total reported daily intake		
**Micronutrient**	**Mean (SD)**	**Median (IQR)**	**Min-max**
Vitamin A (µg)	720 (340)	660 (470-850)	97-2400
Vitamin B6 (mg)	1.8 (0.7)	1.7 (1.3-2.1)	0.4-4.9
Vitamin B12 (µg)	5.2 (2.1)	4.9 (3.6-6.4)	0.9-13
Vitamin C (mg)	110 (61)	97 (63-140)	11-410
Vitamin D (µg)	6.8 (3.4)	6.3 (4.2-8.7)	0.5-26
Vitamin E (mg)	8.8 (3.7)	8.1 (6.1-11)	1.8-28
Vitamin K (µg)	33 (17)	30 (22-41)	3.3-120
Thiamin (mg)	1.3 (0.5)	1.3 (0.9-1.6)	0.2-3.7
Niacin (mg)	16 (5.6)	15 (12-19)	3.8-42
Riboflavin (mg)	1.8 (0.7)	1.7 (1.2-2.1)	0.3-6.1
Iron (mg)	11 (4.9)	10 (7.2-13)	2.2-43
Magnesium (mg)	310 (120)	300 (220-380)	65-770
Potassium (g)	2.9 (1.0)	2.9 (2.2-3.4)	0.68-5.9
Calcium (g)	1.1 (0.44)	1.0 (0.77-1.3)	0.20-3.3
Iodine (µg)	110 (64)	98 (69-140)	10-660
Sodium (g)	2.3 (0.82)	2.2 (1.7-2.8)	0.6-6.9
Folate (µg)	310 (130)	300 (230-380)	43-800
Phosphorus (g)	1.4 (0.49)	1.4 (1.0-1.7)	0.30-3.1
Selenium (µg)	44 (20)	40 (30-51)	6.4-152
Zinc (mg)	9.7 (3.4)	9.4 (7.2-12)	2.3-25
